# Treatment options in stage I seminoma

**DOI:** 10.32604/or.2022.027511

**Published:** 2023-01-12

**Authors:** UROS BUMBASIREVIC, MARKO ZIVKOVIC, MILOS PETROVIC, VESNA CORIC, NIKOLA LISICIC, NEBOJSA BOJANIC

**Affiliations:** 1 Clinic of Urology, University Clinical Center of Serbia, Belgrade, 11000, Serbia; 2 Faculty of Medicine, University of Belgrade, Belgrade, 11000, Serbia; 3 Institute of Medical and Clinical Biochemistry, Faculty of Medicine, University of Belgrade, Belgrade, 11000, Serbia

**Keywords:** Chemotherapy, Radiotherapy, Risk-adapted strategy, Seminoma, Surveillance

## Abstract

Seminomas are most commonly diagnosed in clinical stage I (CSI). After orchiectomy, approximately 15% of patients in this stage have subclinical metastases. Adjuvant radiotherapy (ART) delivered to the retroperitoneum and ipsilateral pelvic lymph nodes has been the mainstay of treatment for many years. Although highly efficient, with long-term cancer-specific survival (CSS) rates approaching almost 100%, ART is associated with considerable long-term consequences, particularly cardiovascular toxicity and increased risk of secondary malignancies (SMN). Therefore, active surveillance (AS) and adjuvant chemotherapy (ACT) were developed as alternative treatment options. While AS prevents patient overtreatment, it is associated with strict follow-up regimens and increased radiation exposure due to repeated imaging. Due to equivalent CSS rates to ART, and lower toxicity, one course of adjuvant carboplatin presents the cornerstone of chemotherapy for CSI patients. CSS is almost 100% for patients with CSI seminoma, regardless of the chosen treatment option. Therefore, a personalized approach in treatment selection is preferred. Currently, routine radiotherapy for CSI seminoma patients is no longer recommended. Instead, it should be reserved for patients who are unfit or unwilling for AS or ACT. Identification of prognostic factors for disease relapse allowed for the development of risk-adapted treatment strategy and stratification of patients in low-risk and high-risk groups. Although risk-adapted policy needs further validation, surveillance is currently recommended in low-risk patients, while ACT is reserved for patients with a higher risk of relapse.

## Introduction

Testicular cancer (TC) is a relatively uncommon tumour, accounting for 1% of all neoplasms in the adult male population. Nevertheless, it is the most prevalent solid malignancy in young men, between the ages of 15 to 40 [1]. While Scandinavian countries continue to have the highest TC incidence, an increasing trend was observed in formerly lower-incidence regions [2]. Accounting for 90%–95% of TC, germ cell tumours (GCT) represent a predominant histologic type, with seminoma comprising about 55%–60% of all GCT [3]. Seminomas that are confined solely to the testis, with no evidence of regional and distant metastases on imaging studies, are classified as clinical stage I disease (CSI). Presumably due to slower progression, approximately 80% of seminomas are diagosed at CSI, compared to 60% in non-seminomatous GCT (NSCGT) [4]. Seminoma is a highly radio- and chemosensitive tumour, with excellent cure rate, reaching 99%–100% for CSI [5]. Although the management of these tumours still remains controversial, it has evolved significantly over the past decades as a result of accumulated evidence enabling a better understanding of long-term therapeutic risks and outcomes.

It is estimated that 15% of patients with CSI seminoma have occult metastatic disease after orchiectomy, typically in the corresponding retroperitoneal lymph nodes [6]. Adjuvant radiation therapy to the retroperitoneal lymph nodes has historically been used to treat CSI seminoma. This therapeutic approach demonstrated great efficacy, with recurrence rates ranging from 1.4% to 6.9%. [7]. However, due to high risk of radiation-associated secondary malignancies, routine use of this treatment modality in an adjuvant setting is no longer recommended by most guidelines [4,8]. Consequently, alternative treatment strategies, active surveillance (AS) and adjuvant chemotherapy (ACT), have been developed. Although cancer-specific survival (CSS) rate for patients on AS is 99%, this treatment strategy has substantial limitations, such as strict follow-up regimen, poor patient compliance, potential risk of secondary malignancies due to excessive radiation exposure from repeated imaging. Furthermore, there is a considerable chemotherapy-induced toxicity burden related to the treatment in case of a disease relapse [9]. On the other hand, routine ACT administration may lead to overtreatment of the patients, increasing the risk of possible long-term morbidity and toxicity.

Irrespective of the chosen treatment modality, CSS for patients with CSI seminoma is virtually 100% [7]. Accordingly, the appropriate selection of treatment strategy has to be based on the comprehensive analysis of the advantages and disadvantages of each treatment option and, equally important, patient characteristics and preferences. The overall impact of treatment burden, cost-effectiveness ratio and quality of life have to be carefully considered in a decision-making model grounded in evidence-based medicine while embracing the balance between professional expertise in the treatment recommendations and patient autonomy in the acceptance of the proposed therapeutic plan.

In this review, we will provide an overview of the available scientific data and current clinical standards in the management of CSI adressing benefits and disadvantages of each treatment option.

## Surveillance

Orchiectomy alone can cure more than 85% of CSI seminoma patients. Given that administration of adjuvant therapy in those patients may lead to overtreatment and subsequent increased short and long-term morbidity, a treatment strategy based on AS has been developed. In order to detect relapse and begin salvage treatment in a timely manner, AS following orchiectomy is characterized by a stringent follow-up strategy that includes regular physical examinations, measurement of serum tumour marker levels, and radiographic assessment. Another argument supporting the use of AS is high chemotherapy and radiotherapy efficacy upon relapse detection.

Relapse rates for CSI seminoma patients on AS have been investigated in multiple studies (Table 1) [10–17]. Most studies had non-randomized comparative design or were based on descriptive series. Reported relapse rates ranged from 12% to 19%. The most prevalent sites of relaps are the retroperitoneal lymph nodes, in 84%–100% of cases, with a median time to relapse between 12 to 18 months. At the time of relapse, 18%–24% of patients had distant metastases [18,19]. Late relapses, most commonly defined as those occurring 2 years after orchiectomy, are rare, and affect 2% of patients on AS, accounting for 9% of all relapses [20]. Computerized tomography (CT) has a crucial role in detection of late relapses. Rarely, a relapse might only present as a clinically palpable disease. In the prospective, single-arm study, which included 88 patients, Choo et al. reported two relapses in ipsilateral inguinal lymph nodes, detected with clinical assessment [15]. On the other hand, in much larger retrospective study from 2014, with 1344 CSI seminoma patients on AS, and relapse rate of 13%, most relapses were initially detected by CT scan (87%) or based on elevation of human choriogonadotropin (3%), while none of the relapses were identified by chest radiography or physical examination [17].

**Table 1:** Active surveillance in stage I seminoma

Author (year)	Patients (n)	Median follow-up (months)	Relapse (%)	CSS (%)	Reference
Duchesne (1990)	113	30	13 (12)	100	[10]
von der Maase (1993)	261	48	49 (19)	98.9	[11]
Chung (2002)	203	110	35 (17)	100	[12]
Aparicio (2003)	143	52	23 (16)	100	[13]
Daugaard (2003)	394	60	69 (18)	100	[14]
Choo (2005)	88	145	17 (19)	100	[15]
Leung (2013)	484	79.2	72 (15)	99.8	[16]
Kollmannsberger (2015)	1344	52	173 (13)	99	[17]

Note: CSS: cancer-specific survival.

The determination of relapse predictors is essential for appropriate patient selection for AS. In a pooled analysis from 2002, which incorporated three large surveillance series, Warde and colleagues identified tumor size >4 cm and invasion of rete testis as significant predictors of disease relapse in multivariate analysis [21]. Moore et al. observed relapse rate of 12.2% in patients with tumour size <3 cm, and 20.3% in patients with tumours >3 cm [22]. Lymphovascular invasion, patient age <34 at the time of diagnosis, and tumour-infiltrating lymphocyte count were identified as possible predictors of relapse in the other studies [20].

The 5-year CSS rate is consistently high across most surveillance studies, with estimated values surpassing 99% [19,20]. Multiple studies compared survival rates between AS and adjuvant treatment (AT) for CSI seminoma patients. In the study from 2013, with data obtained from Surveillance, Epidemiology, and End Results program (SEER) from 1973 to 2003, Jones et al. compared overall survival (OS) and CSS between 5265 patients treated with ART and 1499 patients treated with AS. The OS rates after 5, 10, and 20 years for ART were 97.9%, 95.0%, and 94.8%, respectively *vs*. 92.2%, 83.5%, and 84.1% for AS (*p* = 0.0047), respectively. Additionally, statistically significant difference between AT and AS groups were found for CSS rates for the same observational time-periods (99.6% *vs*. 98.7%, 99.4% *vs*. 98.7%, 99.2% *vs*. 98.7%, *p* = 0.0015). In the multivariate analysis model, ART corelated with improved CSS (HR 0.37, CI 0.20–0.70, *p* = 0.0023) [22]. Glasser et al. also reported improved 10-year OS rate for AT cohort *vs*. AS cohort (95.0% *vs*. 93.4%, HR = 0.58, *p* < 0.0005) in a study which included 33094 patients from National Cancer Database [23]. In another SEER-based study from 2019, with 11206 CSI seminoma patients included, the reported 5-year cancer-specific mortality (CSM) rates were 0.6% *vs*. 0.2% in AS *vs*. AT group of patients (*p* = 0.02). In multivariable analysis, AS was independent predictor of CSM in comparison to AT (HR, 2.59; *p* = 0.04), while it did not affect other-cause mortality (OCM) (HR 1.52; *p* = 0.51) [24].

Current European Association of Urology (EAU) minimal recommendations on follow-up schedule for seminoma patients with stage I disease are based on European Society for Medical Oncology (ESMO) recommendations (Table 2) [8,25]. There is a rising concern over frequent radiation exposure due to repeated CT imaging during the follow-up. The risk of second malignancy related to repeated CT imaging is estimated to be approximately 1 in 300 [26]. Implementation of dose-saving protocols, field limitations, and alternative imaging studies, such as magnet resonance imaging (MRI), could contribute to risk reduction. In a prospective, comparative study from 2020, MRI showed an equivalent diagnostic accuracy compared to CT, with 98% sensitivity for the detection of retroperitoneal lymph node metastases [27]. TRISST was multicentric, randomized, phase III trial, which evaluated noninferiority of MRI or a reduced imaging schedule compared to standard follow-up regimen. The study population consisted of 669 low-risk CSI seminoma patients on AS. Participants were randomly assigned to seven CT scans (6, 12, 18, 24, 48 and 60 months) *vs*. seven MRIs (identical schedule) *vs*. three CT scans (6, 18, 36 months) *vs*. three MRIs (with the same schedule). Primary outcome was defined as 6-year incidence of advanced relapses (≥ stage IIC).

**Table 2:** Recommended minimal follow-up for seminoma clinical stage I on active surveillance

Modality	Year 1	Year 2	Year 3	Year 4&5	After 5 years
Tumour markers and doctor visit	2 times	2 times	2 times	Once	Further management According to survivorship care plan
Chest X ray	–	–	–	–	
Abdominopelvic computed tomography/Magnetic resonance imaging	2 times	2 times	Once at 36 months	Once at 60 months	

Note: From EAU Guidelines. Edn. presented at the EAU Annual Congress Amsterdam 2022. ISBN 978-94-92671-16-5.

Although the event rate was higher in patients with three scans *vs*. seven (2.8% *vs*. 0.3%), that was statistically noninferior. Similarly, MRI was noninferior in comparison to CT, thus indicating that MRI and reduced imaging schedule are not associated with adverse impact on long-term outcomes. The findings of this factorial trial underscored that with surveillance, advanced cases of relapse are rare, salvage treatments are successful, and overall outcomes are favourable, regardless of imaging modality and iteration frequency [28]. Adequate patient compliance is one of the crucial components of surveillance protocols. Unfortunately, there is limited number of studies on this topic, with variable definitions of compliance. Yu and colleagues evaluated compliance in patients with stage I TC within “American private insurance claims” database. Following diagnosis, compliance rate was insufficient with declining rate during longer follow-up periods. Almost 30% of patients did not receive chest and abdominal imaging and serum tumour markers measurement during first year [29]. In another study, after 5.5 years, 21% of AS patients were lost to follow-up [30]. Japanese multicentric study reported that 14.1% of AS patients were lost to follow-up in first two years, and 37.8% during the first 5 years. Predictors of poor compliance were age <36 years and time of diagnosis before 2000 [31]. On the other hand, in a Spanish retrospective small cohort study (n = 64), compliance rate was high in the first year of follow-up (96.8%), while decreasing over time (92.2% after 24 months, and 86.3% after 36 months) [32]. Most series report unsatisfactory compliance rates, thus indicating the need for further improvements in the quality of surveillance protocols, as well as the importance of multifaceted interventions targeting the potential determinants of poor adherence.

One of the disadvantages of surveillance is also the increased uncertainty and concern regarding the higher likelihood of cancer relapse in comparison to adjuvant treatment, with subsequent anxiety and reduction in quality of life. After 10 years of follow-up, nearly 30% of patients reported fear of relapse [9]. Better working ability and higher scores on the satisfaction-of-life scale were reported in AT group in comparison to AS group of patients [33]. Coping ability and level of distress are significant predictors of patient anxiety and should be assessed in the process of treatment planning [9].

Given that all three treatment options for CSI seminoma have comparable survival rates, the cost of treatment must also be taken into consideration in decision-making. Compared to ART, AS results in 39% higher costs per patient over a 5-year follow-up period. Follow-up (predominantly repeated CT imaging) contributes to 91% of all expenses made during surveillance [34]. The cost-effectiveness of AT was compared to AS for CSI seminoma by Cox and colleagues. Although ART and ACT were more cost-effective treatment options, the value of AS was perceived through the possibility of avoiding the overtreatment and associated costs [35]. However, a comprehensive systematic review from 2007. By Groll et al. stated that there is insufficient evidence that AS is more expensive compared to AT [20].

## Adjuvant Radiotherapy

Historically, adjuvant irradiation of retroperitoneal and ipsilateral pelvic lymph nodes (dog-leg configuration) was a standard therapy for stage I seminoma patients. According to most radiation protocols, 25–35 Gy are administered in 15–20 fractions. Due to high seminoma radiosensitivity, long-term CSS after ART are almost 100%, with reported relapse rates between 1% and 6%. In-field recurrences are exceedingly rare, occurring in 0.3% to 1.1% of patients [18]. Most recurrences occur in the first 24 months after irradiation, predominantly in the lungs, posterior mediastinum, left supraclavicular fossa and pelvic lymph nodes. For those patients, salvage radiation or chemotherapy has a 10-year CSS of 97% [19].

Radiation treatment has been associated with substantial long-term consequences and morbidity burden when administered to relatively young patients with a long life expectancy. Due to carcinogenicity of ionising radiation, secondary malignancies (SMN) are a major concern. ART is associated with a two- to six-fold increase in the risk of developing SMN over a 10–15-year follow-up period [36]. In addition, SMN are the major cause of mortality among survivors of testicular cancer [37]. SMN most commonly originate from organs within radiation field, such as stomach, colon, pancreas, bladder, kidney, skin [19]. Multiple studies evaluated the risk of SMN after ART. In a large, retrospective analysis of 40,576 testicular cancer survivors, Travis et al. documented 2,285 SMN during 11.3 years of follow-up. The relative risk (RR) of SMN 10 years after diagnosis was 1.9, while remaining elevated even 35 years after diagnosis (RR = 1.7). There was statistically significant risk elevation for the development of solid cancers (most notably malignant mesothelioma, esophaegal, gastric, colon, pancreatic and bladder cancer). The administration of ART (RR = 2.0, 95% CI = 1.9 to 2.2) or combination of radiotherapy and chemotherapy (RR = 2.9, 95% CI = 1.9 to 4.2) was associated with statistically significant elevated risk of SMN [38]. In a cohort study from 2007, which included 2,707 TC survivors, SMN were detected in 270 (9.9%) patients after 17.6 years of follow-up. ART was associated with a 2.6-fold increased risk of SMN [39]. Horwich et al. reported increased risk of SMN incidence to organs in the field of irradiation. SMN were diagnosed in 468 (17.8%) from total of 2629 CSI patients [40]. Similarly, a large population-based study from 2017. reported that relative risk of SMN after ART was 1.84 in 16,463 seminoma patients from SEER database [41]. A 3-fold increased risk of leukaemia was also linked to ART [42].

Increased cardiovascular morbidity is a well-known long-term adverse effect of radiation exposure. Norwegian comparative study from 2010. reported that RT patients had 2.1-fold greater risk of coronary artery disease (CAD) and 2.3-fold higher risk of an atherosclerotic disease events compared to the surgery group [43]. Cardiovascular events are 2.74 times more likely to occur in CSI seminoma patients receiving ART than among those undergoing AS. Increased incidence of cardiovascular events was observed 5–8 years after ART [44]. Another well-known risk of ART is the gonadal toxicity and impaired spermatogenesis. It is estimated that 2% of total radiation dose is scattered to the contralateral testis, which can result in Leydig cell dysfunction and significant spermatogenesis suppression. Radiation-induced subfertility is reversible within 24 months [45]. Gandini et al. found that 24% of patients had azoospermia 6 months after ART, compared to 6% after 24 months [46]. Prospective Swedish study from 2019. Evaluated total sperm number (TSN) and sperm concentration (SC) in 182 TC patients after ART to para-aortic and ipsilateral pelvic lymph nodes, with a total dose of 25.2 Gy. Transient decrease in mean TSN and SC was observed 6 months after radiotherapy, followed by significant increase in mean TSC and SC 12 months after administration of ART. After 12 months, no significant differences were observed. Authors concluded that adjuvant treatment is not associated with significant deterioration of TSN and SC in CSI TC patients, though, sperm cryopreservation should be offered to patients prior to orchiectomy [47]. ART may also be associated with late gastrointestinal (GI) morbidity presented as peptic ulcer disease, small bowel obstruction, or GI malignancy. In a retrospective review, Hallemeier *et al*. [48] concluded that radiation-induced late GI adverse events are rare, but with high clinical significance [48].

Strategies for minimising radiotherapy toxicity were investigated by multiple randomized trials. In MRC trial from 1999, Fossa and colleagues investigated efficacy and frequency of adverse events associated with RT limited to para-aortic (PA) field (n = 236) *vs*. RT delivered in standard dog-leg configuration (n = 242). After follow-up of 4.5 years, 18 relapses occurred. Each treatment group had 9 relapses. However, all 4 pelvic relapses were detected in PA group. The 3-year OS rate was 100% in dog-leg group and 99.3% in the PA group. PA radiotherapy was associated with reduced frequency of acute toxicities [49]. The efficacy of different radiotherapy doses (20 Gy in 10 fractions for 2 weeks *vs*. 30 Gy in 15 fractions for 3 weeks) was investigated in MRC TE18/European Organisation for Research and Treatment of Cancer (EORTC). Trial involved 625 patients randomly assigned in two treatment groups. After 61 months of follow-up, 10 relapses were detected in 30 Gy treatment group, and 11 relapse in 20 Gy treatment group (HR 1.11). Authors concluded that relapse rates following treatment with 20 Gy are unlikely to be greater than 3% than those following conventional radiotherapy with 30 Gy [50]. Another strategy for reducing toxicity during ART relies on application of gonadal shielding during PA radiotherapy, thus preventing scattered radiation [51].

Since radiation-induced toxicities have been recognized in CSI seminoma patients, the use of ART has declined significantly over the last two decades [52]. Current EAU guidelines do not recommend routine administration of ART in the overall CSI seminoma patient population, but exclusively among those considered unsuitable for AS or ACT [8].

## Adjuvant Chemotherapy

Cisplatin-based chemotherapy regimens are the backbone of the modern therapy for metastatic GCT. Ever since its use has been approved there has been a need to discover chemotherapeutic agent featuring similar efficacy but with improved safety profile [53]. Cisplatin is known to cause several adverse effects including nausea and vomiting, as well as ototoxicity and peripheral neuropathy. In addition, the main dose-dependent side event is renal tubular damage resulting in acute kidney injury or renal insufficiency [54]. Promising findings from preclinical studies indicated that carboplatin was less nephrotoxic and neurotoxic, coupled with decreased emetogenic potency, paved the way for future clinical trials [54,55].

First study to investigate efficacy and safety of carboplatin in adjuvant setting of stage I seminoma was published in 1994. Over the course of 10 years (1982–1992), relapse rates were compared between patients receiving adjuvant radiotherapy (79 patients, median follow-up 51 months), adjuvant carboplatin (78 patients, median follow-up 44 months) and 67 patients undergoing surveillance only (median follow-up 61 months). Among patients receiving carboplatin, 53 had two courses and 25 had one course of adjuvant chemotherapy. Recurrence of 27% was observed on surveillance, 6% in radiotherapy group *vs*. 1% in patients receiving two courses of carboplatin, and none in those receiving one course. Encouraging outcomes of this study lead to conducting randomized clinical trials (RCT) [56].

Between 1996 and 2001, 1477 patients were randomized to receive either adjuvant radiotherapy (para-aortic strip or dog-leg field, 20 or 30 Gy, N = 885) or one course of carboplatin (AUC 7 (area under the curve)—dose of carboplatin calculated as 7 × [glomerular filtration rate − GFR ml/min + 25] mg, N = 560). After median follow-up of 4 years relapse-free survival was similar among two groups at 3 years 95.9% [95% CI 94.4–97.1] *vs*. 94.8% [95% CI 92.5–96.4], respectively. In adition to demonstrated non-inferiority of carboplatin to radiotherapy carboplatin caused fewer acute toxic effects and patients that received this chemotherapeutic compound were less likely to develop second GCT in contralateral testis [57]. Updated results after follow-up of 6.5 years further corroborated previous conclusions. Overall relapse rate was 5.3% in chemotherapy group *vs*. 4% in radiotherapy group [58]. Due to improved safety profile and reduced risk of developing secondary malignancies carboplatin become preferable adjuvant strategy in the treatment of CSI seminoma.

Two more retrospective studies investigated relapse rates after one course of adjuvant carboplatin in the treatment of CSI. Chau et al. analyzed the data of 517 eligible patients in the United Kingdom between 1996 and 2013. and, after median follow-up of 47.2 months 4% of patients had recurrence in retroperitoneal lymph nodes. Median time to relapse was 22.7 months and relapse-free survival after 5 years was 95% [95% CI 92.8–97.3] [59]. Similar results were observed in the Italian cohort. After median follow-up of 22.1 months relapse rate was 5.2% in 115 patients. Investigators reported that carboplatin-related toxicities were manageable ranging from mild fatigue, moderate nausea and vomiting to grade 3 and 4 thrombocytopenia in 5.2% [60].

Following the demonstration of the effectiveness and safety of carboplatin in the adjuvant setting of CSI, the outcome of patients who had relapse was examined. Multicentric retrospective analysis included 185 patients who developed disease recurrence after adjuvant treatment based on one or two courses of carboplatin between 1986 and 2013. The majority of relapses were detected by CT, in 15% of patients based on clinical signs and symptoms and, in 13% the recurrence was assumed after elevation of tumor markers (LDH, Lactate Dehydrogenase and/or βhCG, β-Human Chorionic Gonadotropin). Primary site of recurrence were retroperitoneal lymph nodes (85%). Most relapses (64%) occurred within 2 years after the orchiectomy with the 19 months median time lapse. However, in the present series 15% of cases experienced relapse after more than 3 years and the longest reported time interval from the first diagnosis was 15 years. Cisplatin-based chemotherapy regimen appropriate to the corresponding stage at the time of the relapse was administered in 92% of patients. Second relapse occurred in 28 patients leading to the subsequent chemotherapy or other appropriate treatment modality. After median follow-up time of 53 months, 5-year disease-free survival was 82%, and the 5-year overall survival was 98%. This study concluded that the relapse, after adjuvant carboplatin in CSI, can be successfully treated without imparing the overall survival probabilities, by applying treatment algorithms determined for *de novo* metastatic disease. Furthermore, the authors recommended a prolonged follow-up for patients subjected to adjuvant therapy due to potential late recurrence development [61].

Traditionally, standard dosing of carboplatin was based on skin surface area with 400 mg per square meter. The absence of pharmacokinetic considerations may compromise the precision of such dosage method leading to either elevating systemic exposure to carboplatin, thus causing more carboplatin-related toxicities, or underdosing and diminishing effectiveness. Clinical efficacy and toxicity of carboplatin strongly correlates to glomerular filtration rate (GFR) [62]. Exposure to carboplatin, described as target area under the free carboplatin plasma concentration *vs*. time curve (AUC), is used in equation called “Calvert formula” to calculate individually optimized dose in adults:dose (mg) = target AUC (mg × mL/min) × [GFR (mL/min) + 25 (mg/mL)] [63]. Significance of precise dosing strategy was demonstrated in TE19/EORTC 30982 study. In 347 patients chromium-51 ethylenediaminetetraacetic acid (51Cr-EDTA) measurements of GFR was used, and in 212 GFR was based on 5 creatinine clearance (CrCl), derived from 24 h urine sample. Group of patients whose GFR was based on CrCl received 90% of optimal carboplatin dose and this group had 5-year relapse rate of 7.4% *vs*. 3.9% in 51Cr-EDTA GFR group. Reduction of carboplatin dose by approximately 10% almost doubles the risk for relapse due to decreased therapeutical efficacy [58]. Using the radioactive tracers in everyday practice to determine GFR is not always feasible, and it is associated with elevated method complexity and additional finantial burden [64]. Therefore, GFR estimation formulae were compared to radioisotope GFR measurements in 426 male patients receiving carboplatin. Many formulae were developed for clinical use (Cockcroft-Gault, CKD-EPI, Jelliffe, Martin, Mayo, MDRD, Wright) and these computational models were mainly designed for patients with impaired renal function or older population burdened with comorbidities and malignancies. Significant number of patients would be underdosed if formulas were used: 63%, 49%, and 41% in the case of utilizing Jelliffe, MDRD, or CKD-EPI, respectively. Cockcroft-Gault and Wright equations were more precise leading to underdosing in 18% and 24% of patients, respectively, while Martin and Mayo formulae proved to be the closest to the radioisotope GFR measurements underdosing 9% and 4% of patients, respectively [65].

Carboplatin administration is generally well-tolerated. Most common acute adverse events (AE) include low-grade fatigue, nausea and vomiting occurring in 50% of patients. Another AE is myelosuppression, which typically manifests as neutropenia or thrombocytopenia. Clinical data suggests that life-threating high-grade hematological complications rarely occurre [60]. Long-term side effect are less known. Cisplatin-based regimens are associated with long-term dose-dependent cardiovascular toxicity, as well as ototoxicity, peripheral neuropathy, nephrotoxicity, and development of secondary malignancies [66]. Carboplatin is thought to be less toxic compared to cisplatin-based regimens. After median follow-up of 9 years (range 0.1–20.1 years) in 199 patients that received carboplatin there was no excess in cardiovascular or overall mortality, nor the increased prevalence of secondary malignancies, when compared to general population [67].

Usage of carboplatin in adjuvant treatment of stage I seminoma has been challenged. It was argued that RCT demonstrating carboplatin’s non-inferiority to radiation was robust enough to support such conclusion [68]. Other argument questioning effectiveness of carboplatin referred to its inferiority to cisplatin in advanced seminoma [69]. After 51 patients received four courses of carboplatin to treat metastatic seminoma evidence of disease was present in 18% of patients indicating incomplete response [70]. If carboplatin could not effectively eradicate metastatic disease, was it effective enough in suspected micrometastasis in CSI seminoma? Dieckmann et al. reported higher relapse rates (9.3%) in patients that had know prognostic factor of primary tumor >5 cm and received adjuvant carboplatin [71]. Swedish and Norwegian testicular cancer group (SWENOTECA) compared relapse rates in patients on surveillance and one course of adjuvant carboplatin (AUC7). As expected, surveillance group experienced recurrence more often (4%–19%) than patients receiving AUC7 (2.2%–10.4%) depending on the presence of relevant prognostic factors [72]. The question arises whether the use of carboplatin is justified, having in mind modest benefit over AS in CSI seminoma patients. Similar conclusions could be drawn from several other studies, indicating that additional investigations are warranted to further evaluate the carbplatin role in the contemporary management of CSI [13,72–76].

## Prognostic Factors

In order to identify patients who would benefit from adjuvant therapy it is important to define which patients have a higher risk of disease relapse after surgical treatment of CSI seminoma. The goal is to provide benefit of adjuvant tretment to patients who have a higher risk of recurrence, while minimizing the unnecessary adverse effects hazard in patients whose relapse risk is minimal. Therefore, it is essential to define and validate relevant and convenient prognostic factors.

The first identified prognostic factor was lymphovascular invasion. Back in 1992, Horwich *et al*. [77] followed 103 CSI seminoma patients on AS and after median follow-up of 62 months relapse was diagnosed in 16%. After comparing their primary histological findings lymphovascular invasion was found in 37% of specimens [77]. The size of the primary tumor was the next recognized prognostic factor. Maase et al. followed 261 patients on AS for 48 months, and 19% have relapsed. Among them, 34% had a primary tumor larger than 6 cm in size [11]. In addition to demonstrating that the size of the primary tumor may serve as a prognostic factor, Canadian authors found that the age is a standalone indicator of relapse. In a cohort of 201 patients, those under 34 had a 3.6 times higher risk of recurrence [78]. Prognostic variables were further examined in a pooled analysis. A disease relapse occurred in 19% of the 638 CSI seminoma patients after a median follow-up of 7 years. The primary tumor size >4 cm (HR 2) and rete testis invasion (HR 1.7) have both been identified as statistically significant and independent predictors, particularly when both risk variables were present (HR 3.4) [79]. According to the aforementioned studies, it was postulated that patients with these prognostic indicators should benefit from adjuvant therapy. In contrast, subsequent research failed to validate those findings, arguing that up to 70% of CSI eminoma patients would be overtreated and potentially exposed to the harmful effects if received adjuvant therapy by these criteria [80]. Two more systematic reviews investigated prognostic factors [81,82]. Various studies have been reviewed, leading to the conclusion that primary tumor size is linearly associated with increased risk of disease recurrence, but there is no clear rationale to adopt a specific cut-off value. The assessment of whether rete testis invasion is significant is complicated by the fact that up to 50% of pathologists fail to report or misrecognize it. Furthermore, the variation of reporting featuring lack of distinguishment between pagetoid invasion calls for particular caution in the analysis of pooled pathological data extracted from multiple centres without centralized review prior to interpretation [83].

In an attempt to ameliorate already known prognostic model for the relapse risk in stage I seminoma, several types of biomarkers have been assessed with concerted efforts and a plethora of potential biomarkers have been identified (Table 3). Ideally, a single prognostic biomarker, or preferably a panel of certain biomarkers, required for more precise risk stratification should be sensitive and specific enough to identify a stage I seminoma patient with high-risk designation. In addition to commonly obtained samples (blood, urine, or tissue samples), other sample sources or even collection sites (e.g., testicular vein) can be considered in further prognostic biomarker studies. Conventional techniques along with routine laboratory practice are usually employed in such studies. However, common detection techniques are being gradually substituted by Next Generation Sequencing (NGS) technologies, as well as Multiomics techniques that are not readily available and require translation “from bench to bed side”.

**Table 3:** Prognostic factors for disease relapse after orchiectomy for patients with stage I seminoma

Type of marker	Assessed markers	Sample	Detection technique	Main findings	References
Derivative	Inflammatory indices (NLR, dNLR, PLR, SII, SIRI, LMR)	Blood	Routine laboratory practice	The difference between the median values of certain inflammatory indices in stage I and advanced stage were statistically significant	[88,89]
Proteins	Markers related to proliferation and to the surrounding immune microenvironment	Orchiectomy tissue specimens	Immunohistochemistry	Although some candidate biomarkers (CXCR4, PD-1/PD-L interaction, etc.) indicated promising application, further research is needed to optimize patient’s risk stratification	[90]
Nucleic acids	Non-coding RNAs miR-371a-3p	Blood (serum)	RT-qPCR diagnostic kit	Not prognostic for relapse, however, may serve as an early marker of relapse	[91,85]
Signature of alterations	Altered gene expression signature	Orchiectomy tissue specimens	Gene expression analysis	A discriminating signature for relapse was not identified	[92]
	Altered protein expression signature	Orchiectomy tissue specimens	Quantitative proteomic approach	Significant difference in protein expression levels of filamin A, PARK7 and 14-3-3γ was associated with rete testis invasion and may help to identify patients with poor prognosis	[93]	

Note: NLR—neutrophil-to-lymphocytes ratio; dNLR—derived neutrophil-to-lymphocyte ratio (formula: neutrophil/(WBC-neutrophil)); PLR—platelet-to-lymphocyte ratio; SII—systemic immune-inflammation index (formula: neutrophil × platelet/lymphocyte); SIRI—systemic inflammation response index (formula: neutrophil × monocyte/lymphocyte); LMR—lymphocyte-to-monocyte ratio; CXCR4-C-X-C chemokine receptor type 4; programmed-death-1 receptor (PD-1) and its ligand (PD-L1); RNA- ribonucleic acid; miR- micro RNA.

In order to establish sensitive, specific, stable, convenient, minimally invasive, and cost-saving biomarkers that could bring additional value for the risk assessment of relapse in stage I seminoma, further studies, preferably with well-defined methodology, should be carried out before sound recommendations with clinical robustness can be made. The closest to clinical application in this particular scenario are certainly small non-coding RNA molecules: micro RNAs (miRs) [84,85]. The role of this type of biomarkers is currently being evaluated in several clinical trials, recruiting men diagnosed with CSI seminoma [85]. Hopefully, such trials will provide a uniform pipeline of miRs for analysis, further refinement and, eventually, clinical application.

Knowledge of prognostic factors could significantly improve the management of stage I seminoma. Although primary tumor size can be viewed as a continuous variable associated with increased risk of disease recurrence (larger tumors are associated with higher risk), there is no level of evidence for other predictors to support their routine clinical application.

## Risk-Adapted Treatment

In a risk-adapted approach, patients are divided into groups with low and high-risk of disease recurrence, based on the presence of prognostic factors. The idea on how to stratify patients was evolving over the course of time, aiming to avoid overtreatment and unnecessary exposure to potential toxicities of adjuvant therapy, and, on the other hand, to reduce the risk of even more toxic therapy in disease recurrence or cumulative radiation dose during CT scans on surveillance [86,87].

During 5-year period (1994–1999) 203 CSI seminoma patients with a median follow-up time of 52 months were divided into two groups, based on the presence of risk factors for worse prognosis (presence of histological tumor stage pT2 or higher and/or lymphovascular invasion). Those regarded as low-risk (70.4%) underwent surveillance and 29.6% of patients, who were considered high-risk, received two courses of carboplatin. Relapse rates in the carboplatin group were 3.3% *vs*. 16.1% in the surveillance group, with 5-year disease-free survival favoring carboplatin (96.6% *vs.* 83.5%) [11]. Those results were promising, but the choice of prognostic indicators was not based on previously validated data. The same group of authors redesigned the protocol after Warde et al. proposed primary tumor >4 cm and rete testis invasion as prognostic factors [73]. After median follow-up of 34 months, 314 CSI seminoma patients received two courses of carboplatin (68.2%) if either or both risk factors were present or otherwise underwent AS. CSS was 100%, and relapse rates in AS group were reduced to 6% compared to the previous investigation, but rather high proportion of patients (68.2%) received adjuvant chemotherapy. Considering the results of the previous research, the following study carried out risk stratification based on the same criteria but favoring adjuvant carboplatin only if both prognostic factors were present. Including 214 CSI seminoma patients, 33% received chemotherapy, among whom 1.4% developed relapse *vs.* 9.8% of patients on AS [74]. In conclusion, 67% of patients did not receive adjuvant chemotherapy and majority of high-risk cases avoided disease recurrence. Apart from previous study, the fourth one by the Spanish Germ Cell Cancer Group maintained the same design and only included the existence of rete testis invasion as a predictive factor of a poorer outcome [75]. Invasion of rete testis was present in 47.4% of 135 CSI seminoma patients, and they received adjuvant carboplatin. The 3-year disease-free survival rate for all patients was 94.9% (92% for AS and 98.2% overall). Although excellent results were obtained when only one prognostic indicator was taken into account, additional research is required to make the stratification even more successful.

The SWENOTECA trial considered tumor size >4 cm and rete testis invasion as prognostic indicators, however in addition to recommending AC in high-risk patients, the patients’ attitudes and autonomy were also taken into consideration, so they were free to select AS or AC after surgical treatment for CSI seminoma [72]. Research included 1118 patients, 422 on AS and AC was administered to 690 patients. Interestingly, even though only 11.2% of patients had both risk factors, 53% decided to receive AC. Relapse rates between groups were comparable (7.5% for AS and 6% for AC), with significant results obtained after risk stratification within groups. Among patients managed by surveilance, 4% were diagnosed with recurrence in the absence of prognostic factors, compared to 15.5% in patients displaying one or two predefined risk indicators. In AC group, the reported relapse rates of 2.2% and 9.3% were observed, depending on the absence or presence of risk factors. Results of studies which compared ACT *vs*. AS are summarized in Table 4.

**Table 4:** Risk-adapted treatment based on prognostic factors

Author (year)	Patients (n)	High risk *vs*. Low-risk (%)	Median Follow-up (months)	Relapse rate ACT *vs*. AS (%)	Desease-free survival ACT *vs*. AS (%)	Reference
Aparicio (2003)	203	29.6 *vs*. 70.4	52	3.3 *vs*. 16.1	96.6 *vs*. 83.5	[13]
Aparacio (2005)	314	68.2 *vs*. 31.8	34	3.3 *vs*. 6.0	96.2 *vs* 93.4	[73]
Aparicio (2011)	214	33.0 *vs*. 67.0	34	1.4 *vs* 9.8	98.0 *vs* 88.1	[74]
Aparicio (2018)	135	47.4 *vs*. 52.6	33	1.6 *vs*. 7.0	98.2 *vs*. 92.0	[75]

Note: ACT: adjuvant chemotherapy; AS: active surveilance.

The future of CSI seminoma patients management should be guided by prognostic criteria and stratification depending on the risk of disease relapse. External validation of prognostic indicators is needed, as well as a search for more effective adjuvant therapeutic options than carboplatin.

## Conclusion

Stage I seminoma is highly curable disease regardless of the chosen treatment modality. As a result, selecting the appropriate adjuvant therapy remains a controversial issue. Although highly efficacious, routine adjuvant radiotherapy is no longer recommended, primarily due to long-term risk of secondary malignancies. While surveillance prevents patient overtreatment, it is associated with strict follow-up regimens and increased radiation exposure due to repeated imaging. On the other hand, routine ACT administration may lead to overtreatment of the patients, increasing the risk of long-term morbidity and toxicity. Accordingly, selection of appropriate treatment strategies should be based on individualized approach, with careful consideration of all the advantages and disadvantages of each treatment modality, concomitantly acknowledging patient preferences and autonomy. Although current risk-based treatment is helpful in clinical practice, further studies are required for validation of the established and identification of new prognostic factors.
